# Status of cancer education in middle and high schools in southern Saudi Arabia: An exploratory descriptive study

**DOI:** 10.1097/MD.0000000000048793

**Published:** 2026-05-15

**Authors:** Aziza Alshahrani, Zahrah Asiri, Shahad Alahmari, Saja Alasmari, Malak Alsultan, Dina Alqahtani, Reem Alqahtani

**Affiliations:** a Department of Pharmacology, College of Pharmacy, King Khalid University, Abha, Saudi Arabia; b Department of Pharmaceutics, College of Pharmacy, King Khalid University, Abha, Saudi Arabia; c College of Pharmacy, King Khalid University, Abha, Saudi Arabia.

**Keywords:** cancer education, health literacy, health promotion, Saudi Arabia, teacher perspectives

## Abstract

Cancer incidence is increasing in Saudi Arabia, and in the Asir region, many cases are diagnosed at advanced stages. Schools are often considered advantageous settings for early prevention, but little is known about how cancer education is currently addressed or perceived within school settings. This exploratory study aimed to assess the status of cancer education in middle and high schools in the Asir region by examining science teachers’ practices, perceived barriers, and views on the feasibility of integrating cancer-related topics into routine instruction. An exploratory descriptive study was conducted with 22 science teachers from 5 urban public schools in the Asir region using structured interviews that included yes/no and open-ended questions. Descriptive statistics summarized closed-ended responses; open-ended data were analyzed through manual inductive content analysis. While most teachers reported including some cancer-related content in their teaching, coverage was inconsistent and typically breast cancer-focused. Only 2 teachers had received training on cancer education. Barriers included a lack of institutional support, time constraints, and beliefs that cancer education was outside their teaching responsibilities. Teachers generally viewed cancer education as important and feasible, with 20 of 22 expressing willingness to receive training and all participants supporting school–health partnerships such as guest speakers. Concerns about potential student anxiety were mixed: 13 of 22 teachers felt cancer education would not cause anxiety, whereas 9 of 22 were concerned that it might. Although based on a small, convenience-based sample drawn only from urban public schools, this study provides preliminary insight into how science teachers in 1 region of Saudi Arabia engage with cancer education. Findings highlight fragmented implementation, limited training, and considerable teacher interest in improving school-based programs. These results suggest the value of further research to better understand cancer education in school settings.

## 1. Introduction

In Saudi Arabia (SA), the incidence of cancer has been steadily increasing, presenting a significant public health challenge.^[1,2]^ In regions such as Asir, located in southern SA, many patients are diagnosed at advanced stages, a trend partly attributed to limited public awareness and delayed screening.^[3–6]^ Given that a substantial proportion of cancer risk is tied to modifiable behaviors, including tobacco use, poor dietary habits, and sedentary lifestyles,^[7,8]^ early education is widely recognized as a critical component of comprehensive cancer prevention.^[9–11]^ Furthermore, national studies have documented prevalent public misconceptions regarding carcinogens and cancer risk factors,^[12]^ underscoring an urgent need for structured, evidence-based health literacy initiatives.

Schools represent a strategic and highly advantageous setting for this early intervention.^[13]^ Adolescence is a formative developmental stage during which individuals establish long-term lifestyle habits that directly influence their future disease risk.^[14]^ While short-term, campaign-based interventions in SA have successfully improved student knowledge and attitudes regarding targeted topics, such as breast cancer and human papillomavirus,^[15,16]^ the impact of ad hoc programs is often difficult to sustain.^[17]^ Integrating cancer-related health promotion directly into routine schooling provides the continuous, structured exposure necessary to support lasting behavioral change.^[18]^ Within this environment, middle and high school science teachers are particularly well-positioned to serve as key facilitators of preventive education. Through their standard curriculum covering human biology, cells, and genetics, science teachers have repeated opportunities to address health misconceptions and reinforce preventive behaviors.^[14]^

At the national level, Saudi Vision 2030’s Health Sector Transformation Program and the Ministry of Education’s Health-Promoting Schools initiative prioritize health promotion and disease prevention within school settings, recognizing schools as key platforms for improving population health.^[19–21]^ Despite this clear national ambition, there is limited empirical information on how cancer-related topics are currently addressed in the classroom.

Therefore, this exploratory study aimed to describe the current status of cancer education in Asir middle and high schools by examining science teachers’ existing practices, perceived barriers, school-level initiatives, and views on the feasibility of integrating cancer-related topics into their routine instruction. Importantly, this study focuses strictly on teachers’ perceptions and self-reported classroom practices; it does not include an independent review of official Ministry of Education curriculum documents, and thus should not be interpreted as a formal evaluation of national educational policy.

## 2. Methods

### 2.1. Study design, setting, and participant selection

An exploratory descriptive study with qualitative elements was conducted to examine the status of cancer education in middle and high schools in the Asir region. A total of 22 teachers were recruited from 5 urban public schools in the region. These teachers taught chemistry, biology, anatomy, and science courses. Data were collected on the subjects they taught and the grade levels they covered. The inclusion and exclusion criteria for the teachers are presented in Table 1.

**Table 1:** Inclusion and exclusion criteria.

Inclusion criteria	Exclusion criteria
Teach science, biology, chemistry, physics in middle and high school in Asir region.	School administrators, counselors, and other nonteaching personnel. Teachers who do not teach science related subjects.

Schools were selected using convenience sampling based on geographic proximity and preexisting relationships with staff (e.g., alumni connections or community ties of the research team). The research team approached 8 urban public schools. Of these, 5 agreed to participate and were able to accommodate the interviews within the study timeframe; the remaining 3 could not be included because access would have required additional authorization from the Asir Education Directorate, which could not be obtained within the available study period. Private schools and schools in rural areas were not approached in this study. We did not have access to a complete list of eligible schools or teachers in the region, and therefore could not estimate the total number of potentially eligible participants or calculate a response rate.

The study size was determined pragmatically, based on the number of teachers accessible during the study period, with the intent of generating initial exploratory insights into cancer education. While convenience sampling limits generalizability, it is methodologically appropriate for exploratory research aimed at identifying preliminary patterns and generating hypotheses rather than making population-level inferences. Consequently, statistical representativeness was not pursued in this study.^[22]^ No a priori sample size calculation was performed, and data saturation was not formally assessed. Given the small, convenience-based sample and the brief, often repetitive nature of responses, it is likely that not all relevant views or categories were captured. As such, the sample reflects only a narrow, accessible subset of the broader school system, which limits the transferability of our findings beyond the studied group, and the findings are best interpreted as descriptive and preliminary, intended to inform future, more comprehensive studies.

### 2.2. Data collection procedures

A 10-question structured interview was conducted face-to-face with the science teachers. The interview explored 3 key aspects: existing cancer education initiatives, the importance of cancer education, and the feasibility of integrating cancer-related topics into routine instruction.

Interviews were conducted in Arabic and lasted approximately 15 to 20 minutes each. The interview guide, designed and organized in Google Forms, included both yes/no and short-answer questions. Interviews were conducted by undergraduate pharmacy students from the research team.

Interviews were not audio-recorded. Instead, responses were typed directly into a Google Form during each session. We acknowledge this as a methodological limitation: real-time note capture by student interviewers carries a risk of truncation, paraphrasing, and selective capture, and some nuance and detail may have been lost.

### 2.3. Translation and data analysis

Short-answer responses were later translated into English by bilingual members of the research team. One researcher produced the initial translation, and a second bilingual researcher reviewed all translations against the original Arabic. Any discrepancies were resolved by discussion; approximately 20% of the responses required such discussion to reach consensus. No formal back-translation or semantic equivalence check was performed. We acknowledge this as a limitation of the translation process, as subtle shifts in meaning between the original Arabic responses and their English rendering cannot be ruled out.

Yes/no responses were summarized using frequency distributions. Open-ended answers were analyzed using manual inductive content analysis.^[23]^ Each student researcher first independently read all responses and generated an initial set of descriptive codes close to the wording used by teachers. After this independent coding, the researchers compared their codes and discussed discrepancies until a consensus was reached. Codes that referred to similar content were then grouped into broader descriptive categories under faculty supervision, and category labels and boundaries were iteratively refined until each category was internally coherent and clearly distinguishable from the others. We did not attempt to develop higher-order interpretive themes or theoretical constructs from the data, as the brief, note-based nature of the responses supports descriptive categorization rather than deeper interpretive analysis. Voyant Tools was used only to generate a word cloud of frequent terms (https://voyant-tools.org/).^[24]^

### 2.4. Ethical considerations

Ethical approval for the study was obtained from the research ethics committee at King Khalid University (ECM#2025-502). Informed consent was obtained from all participants prior to the interview, and the participants and their schools were anonymized.

## 3. Results

### 3.1. Participant characteristics

Participating teachers (n = 22) were drawn from 5 schools. As shown in Table 2, 14 teachers (64%) were female, and 8 (36%) were male. Thirteen teachers (59%) taught high school, and 9 (41%) taught middle school. Regarding the courses they taught, 9 teachers (41%) taught science, 7 (32%) taught biology, 5 (23%) taught chemistry, and 1 (5%) taught physics.

**Table 2:** Demographic and teaching characteristics of participating teachers (n = 22).

Characteristic	Count (n = 22)	Percentage
Gender		
Female	14	64
Male	8	36
School level		
High school	13	59
Middle school	9	41
Subjects taught		
Science	9	41
Biology	7	32
Chemistry	5	23
Physics	1	5

### 3.2. Teacher responses

As described earlier, the interview participants were asked about 3 domains related to cancer education in schools. The interview guide was adapted from a previously published study,^[25]^ with the addition of questions to explore further perspectives specific to our research context.

### 3.3. Existing cancer education initiatives in Asir schools

**Figure 1.**
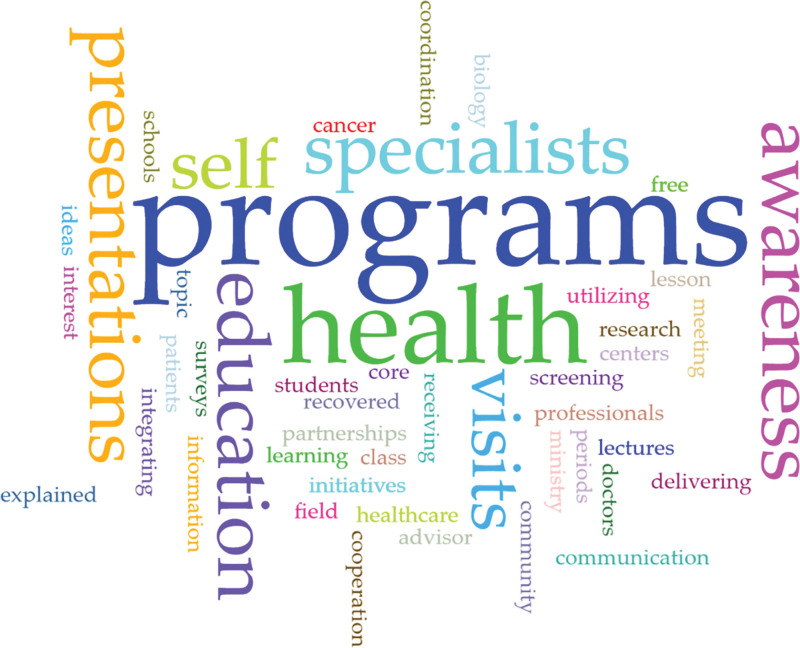
Word cloud of teachers’ suggestions for enhancing school-based cancer awareness programs. The figure provides a visual complement to the manual content analysis of teachers’ open-ended responses. Word size reflects frequency of occurrence, helping to illustrate emphasis on concepts such as “programs,” “health,” “education,” “visits,” “presentations,” “awareness,” and “specialists.”

When teachers were asked about their personal inclusion of cancer education in teaching, 5 of 22 teachers (23%) stated that they do not incorporate it (Table 3). The reasons provided included a lack of time, the fact that cancer education is not originally part of the curriculum, and the belief that it should be restricted to biology courses. Conversely, 17 of 22 teachers (77%) reported that they do incorporate cancer education in their teaching. These teachers were asked to describe their efforts in more detail, and through content analysis, their responses were categorized into 4 main categories: curriculum-based teaching (covering only what is originally included in the curriculum), raising awareness about early detection, educating about cancer risk factors, and promoting general cancer awareness (Table S1, Supplemental Digital Content). In addition, when asked if their school takes any measures to incorporate cancer education (Table 3), 7 of 22 teachers (32%) stated that their school does not, with some attributing this to a lack of resources. In contrast, 15 of 22 teachers (68%) reported that their school does take measures to include cancer education. However, content analysis showed that these activities were rarely comprehensive. Six of the 15 teachers explicitly described breast cancer-focused initiatives (e.g., breast cancer campaigns, partnerships with clinics, or lectures specifically about breast cancer). The remaining 9 teachers cited other efforts, primarily referring to general awareness days, one-off seminars, or unspecific “programs and activities” rather than structured, multi-cancer curricula (Table S2, Supplemental Digital Content). Taken together, this pattern indicates that, in this sample, school-level “cancer education” was predominantly operationalized as breast cancer awareness activities, with limited evidence of broader, multi-cancer educational approaches. In addition, we asked teachers how cancer awareness programs in schools could be improved. Their open-ended responses were first analyzed manually using content analysis to identify recurring ideas and insights. To visually complement this content analysis, we used Voyant to generate a word cloud (Fig. 1). The visualization highlights the emphasis teachers placed on structured cancer education programs, visits, and presentations by specialists. Finally, when we asked teachers whether they had ever received training in teaching cancer-related topics, only 2 reported having undergone such training (Table 3). The remaining responses highlighted barriers that can be broadly classified into 2 main categories: structural constraints and individual-level factors. Structural constraints included a lack of coordination or institutional support (n = 3), a lack of opportunity or access (n = 3), and a lack of resources (n = 4). Individual-level factors included the perception that the topic was not within their subject scope (n = 3), low personal interest (n = 1), and the perceived sufficiency of the existing curriculum (n = 1; Table S3, Supplemental Digital Content).

**Table 3:** Teachers’ perspectives on cancer education in schools, grouped by domain (existing initiatives, importance, and feasibility).

Domain	Variable	Yes (%)	No (%)
Existing cancer education initiatives	Do you personally incorporate cancer education into your teaching?	77% (17)	23% (5)
	Does your school have any programs or initiatives that integrate cancer awareness into the curriculum?	68% (15)	32% (7)
	Have you ever received training on teaching cancer-related topics?	9% (2)	91% (20)
Importance of cancer education	Do you believe cancer education in schools would be beneficial?	100% (22)	0% (0)
	Do you think raising awareness might increase students’ anxiety?	41% (9)	59% (13)
	Do you think cancer awareness has an impact on students’ mental well-being?	64% (14)	36% (8)
Feasibility of integrating cancer topics	Do you believe incorporating cancer topics into the curriculum would be feasible?	95% (21)	5% (1)
	Would you be willing to take training on teaching cancer-related topics?	91% (20)	9% (2)
	Do you think guest speakers would be beneficial?	100% (22)	0% (0)

### 3.4. The importance of cancer education

We next asked the teachers whether they believed that cancer education in schools would be beneficial, and all participants agreed on the importance of including cancer education in schools (Table 3). In addition, teachers were asked to provide a brief explanation of why they believe cancer education is important. Content analysis of their responses revealed 2 major categories: the first emphasized promoting basic cancer knowledge among students, with the potential for this awareness to extend to their families; the second focused on encouraging preventive behaviors and raising awareness about the importance of early detection (Table S4, Supplemental Digital Content). Furthermore, teachers were also asked whether they thought raising awareness about cancer might increase students’ anxiety about their health. While 13 teachers (59%) responded that it would not cause anxiety, 9 teachers (41%) expressed concerns that cancer education could potentially lead to heightened health-related anxiety among students. In addition, teachers were asked if they believed that cancer awareness has an impact on students’ mental well-being. A total of 14 of 22 (64%) indicated that cancer awareness influences students’ mental well-being, whereas 8 of 22 (36%) felt it does not have a significant impact (Table 3).

### 3.5. Feasibility of integrating a cancer education curriculum in classrooms

Lastly, we asked teachers whether they believed integrating cancer-related topics into the routine instruction would be feasible in their classroom or school. A total of 21 of 22 teachers responded that it would be possible. Only 1 teacher expressed a different view, suggesting that external programs are available for students to join and learn from instead (Table 3). We asked them why they think incorporating cancer in their routine instruction was feasible, and by content analysis, we identified 4 categories. The first category is that it is feasible because it is already in the curriculum. This category reflects responses indicating that there are existing lessons about cancer-related topics, such as cell division, mutations, and body systems, particularly within science, biology, and chemistry courses. The second category, is feasible because it is necessary to raise awareness and health education, as reflected in teachers’ views that integrating cancer education would enhance students’ general awareness of diseases and promote health-related discussions within the classroom. The third category, community benefit and prevention, captured responses emphasizing the role of cancer education in fostering preventive behaviors and contributing to a healthier society by informing students at an early age. The fourth category, understanding the disease and its causes, included responses highlighting the importance of providing students with knowledge about cancer’s causes and ensuring access to accurate information that could benefit both students and their families. These categories demonstrate the range of practical and educational reasons teachers identified to support the feasibility of integrating cancer education into existing school curricula (Table S5, Supplemental Digital Content). We asked the teachers whether they would be willing to take courses or training on teaching cancer-related topics, and 20 of 22 teachers (91%) responded that they would (Table 3). All of them indicated that it would be beneficial, particularly in helping them raise awareness. Two teachers, however, expressed reluctance, explaining that their age might pose a challenge to participating in such training. In addition, we asked teachers whether they believed that hosting guest speakers from health sciences colleges to talk about cancer, along with providing resources and activities for students, would be beneficial. All participants agreed that such initiatives would be valuable (Table 3). Moreover, in response to the open-ended question “what is your opinion and suggestion about cancer programs?”, teachers generally emphasized the need for systematic, well-organized cancer education initiatives rather than ad hoc activities.

They advocated for practical components to make cancer education more tangible, such as utilizing morning school broadcasts and leveraging events like World Cancer Day to consistently reinforce health messages. To foster active engagement, several teachers recommended having students conduct independent research and deliver presentations. Furthermore, participants proposed integrating real-world contexts by inviting cancer survivors to share their recovery journeys, hosting medical specialists, and organizing visits to local health centers. Throughout these suggestions, teachers consistently underscored the importance of positive messaging focused on early detection, aiming to educate students without inciting unnecessary fear.

## 4. Discussion

This study focused on school-based settings because schools are often considered advantageous sites for health promotion. Children spend a large proportion of their time in the classroom, making schools frequently described as venues for sustained educational interventions.^[26,27]^ Health organizations emphasize that health education is an integral part of schools’ mission: it imparts the knowledge and skills young people need to be healthy adults.^[28]^ The importance of early preventive education is underscored by the growing cancer burden. In SA, cancer incidence is projected to rise substantially, with an estimated 60,429 new cases by 2040, up from 27,885 in 2020 (+116.7%).^[1,2]^ In 2015, the yearly economic burden, including treatment and productivity losses, was estimated at US $7.9 billion, with projected cumulative costs hitting approximately US $159 billion between 2015 and 2030.^[29]^ Cancer is also among the leading causes of premature mortality and disability in SA, contributing substantially to years of healthy life lost.^[30]^ At the same time, evidence suggests that a substantial proportion of cancers, along with most cardiovascular diseases and diabetes, could be prevented through effective health education.^[27]^

Thus, integrating cancer education into the formal science curriculum has the potential to reach many adolescents in a more systematic way than ad hoc methods. Our findings showed that, in practice, cancer-related content was included inconsistently and depended on individual teacher initiative. Without perceived formal policy support or curriculum alignment, even motivated teachers found efforts fragmented and unsustainable.^[26]^ In the Asir region, this fragmented approach is particularly concerning, as a previous study reported that 20.8% of newly diagnosed cancer patients presented with metastasis, and among these, 77.8% were diagnosed at stage IV.^[31]^

Teachers in our sample overwhelmingly equated “cancer education” with breast cancer, consistent with the common global focus on breast cancer awareness. While breast cancer is indeed important, this narrow framing carries the risk of 2 critical educational consequences. First, it may foster a selective pattern of risk awareness: students gain relatively detailed knowledge about breast cancer symptoms and self-examination, while remaining poorly informed about shared, modifiable risk factors such as physical inactivity, obesity, tobacco use, and human papillomavirus that cut across multiple cancers.^[32–34]^ In practice, this means that “knowing about cancer” is equated with recognizing 1 disease and a narrow set of behaviors, rather than understanding a broader constellation of preventable risks. This pattern is consistent with regional data from Asir, where awareness of colorectal cancer symptoms, risk factors, and screening methods remains low, as does knowledge about thyroid cancer.^[5,35]^ Second, positioning breast cancer as the primary focus of school “cancer topics” has important implications for gendered disease perception.^[36,37]^ Educational and public-health messaging typically frames breast cancer as a women’s disease, delivered predominantly to girls and women. Indeed, reviews of educational interventions show that few explicitly address male breast cancer or involve male participants.^[38]^ When health education relies heavily on feminized narratives, such as pink-ribbon campaigns, it can reinforce a gendered disease schema in which “cancer risk” becomes associated primarily with female bodies. This school-level framing mirrors a broader societal pattern where awareness of male vulnerability to breast cancer remains low; for instance, 1 study found that over 60% of surveyed adults did not know men could develop the disease.^[39]^ Furthermore, qualitative and quantitative evidence suggest that this feminized framing creates stigma, surprise, and a sense of “misfit” when men receive a breast cancer diagnosis, which can delay clinical diagnosis and support-seeking.^[39,40]^ Within a school context, a critical educational consequence of this gendered schema is the risk of cognitive and participatory disengagement among male students. If boys encounter cancer prevention predominantly through feminized content, they may infer that the topic does not apply to them. According to the health belief model (HBM), perceptions of personal susceptibility are central to motivation.^[41,42]^ Therefore, this narrow framing not only obscures male vulnerability to breast cancer, but may also lead boys to underestimate their own risk for other cancers. Making school-based cancer education more inclusive of both sexes and multiple cancer types would likely support more balanced risk awareness and prevention. Although our data cannot speak directly to differences between girls’ and boys’ schools, since our sample was not designed for that comparison, the broader literature on SA’s gender-segregated school system describes differential emphases in how health topics are addressed. Prior work suggests that breast cancer awareness has been emphasized more in girls’ schools, with boys having fewer structured opportunities to engage with the topic, linked to the local cultural context.^[43]^ Consistent with this literature, studies of male high school students in SA have reported limited breast cancer knowledge.^[44]^ This early educational disparity may contribute to a gender gap that is also observed in adulthood. Recent regional data strongly support this; a cross-sectional study of university students and employees in the Asir region found that females had over twice the odds (odds ratio = 2.01) of possessing adequate breast cancer awareness compared to males.^[33,44]^ Furthermore, this regional data aligns with broader national adult trends, where far fewer Saudi men (19%) than women (24%) report knowledge of breast cancer screening.^[45]^

One mechanism proposed in the literature for this gap relates to cultural and social sensitivities – rather than religious doctrine – that often discourage the discussion of body-related topics across genders, with the consequence that both boys and girls may receive limited information about health issues primarily affecting the opposite sex.^[46]^ One element that emerges from our data is that teachers’ worries about student anxiety may also reflect awareness of parental influence over school-based health discussions, although this interpretation is tentative given the brief nature of teachers’ responses on this point.

In our sample, all teachers agreed that including cancer education in schools would be beneficial and generally viewed schools as a practical setting for health promotion, echoing international evidence that school-based programs can influence youth behaviors.^[9]^ However, some also expressed reservations, for example, concerns that such education might increase students’ anxiety and statements that cancer topics were “not within my field” or “not my specialty,” indicating uncertainty about whether they themselves should be responsible for delivering this content. These views reflect teachers’ perceptions rather than observed or measured outcomes. When interpreted through established health education theories, teachers’ comments align conceptually with constructs described in social cognitive theory and the HBM. For example, references to awareness, role modeling, and prevention echo social cognitive theory concepts related to social learning and self-efficacy, while discussions of risk awareness and prevention parallel HBM constructs such as perceived susceptibility and perceived benefits.^[42,47]^ These theories were applied post hoc to categorize teachers’ perspectives, not to guide the interview or analysis.

In terms of delivering cancer content to students, teachers in our data did not specify exact time allocations, but several described linking cancer topics to existing lessons (e.g., cell division, mutations, or human body systems), suggesting that integrating cancer content into current science units may feel more feasible than adding separate sessions. In their suggestions for cancer programs, teachers often emphasized practical, hands-on elements such as school-based activities, demonstrations, or real-life case discussions, and many highlighted guest sessions led by university or health professionals as a helpful way to bring specialized expertise into the classroom. These ideas reflect teachers’ perceptions of feasibility and would need to be refined and aligned with evidence-based school health education standards and relevant ministry guidance in any future program design.^[20,48]^

Despite their enthusiasm, few teachers reported receiving formal training in cancer education. This gap in preparation mirrors findings from other school health programs: without specific teacher training and resources, integration of new health topics is limited.^[26]^ In our data, teachers identified a range of barriers to cancer education training and implementation. Structurally, several respondents cited a lack of official directives or nomination from the Ministry of Education, with comments such as “not required by the Ministry.” Logistical constraints were also evident, including time limitations and a crowded curriculum, as expressed in remarks like “I didn’t get the opportunity.” Some barriers reflected teachers’ perceptions of their professional roles; participants noted that cancer education was “not within my field” or “not my specialty,” suggesting a belief that the topic falls outside their instructional responsibilities. Finally, a few teachers indicated low personal motivation or interest, offering responses such as “not interested” or “we just teach what we have.” These varied explanations underscore the need for multifaceted strategies that address both systemic and individual-level barriers to implementation.

Although our analysis was inductive and was not guided by a predetermined framework, the barriers described by teachers can be read alongside established implementation science frameworks such as the Consolidated Framework for Implementation Research and the Reach, Effectiveness, Adoption, Implementation, and Maintenance framework.^[49,50]^ The lack of in-school training and materials broadly corresponds to what Consolidated Framework for Implementation Research calls inner-setting determinants, while teachers’ references to the absence of a Ministry directive map onto its outer-setting determinants.^[49]^ Reach, Effectiveness, Adoption, Implementation, and Maintenance similarly highlight adoption, implementation, and maintenance as distinct dimensions that each require attention during program rollout, including through training and sustained policy support.^[50]^

Concerns about student anxiety were mixed among teachers. A few anticipated that talking about cancer might increase fear, while most believed that, handled properly, education could reassure students. These are teachers’ perceptions of hypothetical student reactions; we did not collect data from students themselves. Prior research similarly finds both risks (heightened worry) and benefits (empowerment) in adolescent health education.^[51]^ This underscores the need for sensitive content design: emphasizing prevention, success stories (survivorship), and actionable steps tends to empower students and reduce fatalism. Age-appropriate framing and positive role models can mitigate anxiety (e.g., highlighting advances in treatment and lifestyle prevention, rather than just disease).^[51]^

Nearly all teachers were eager to receive help from health professionals. They welcomed ideas like guest speakers from universities or clinics to bring expertise into classrooms. This suggests strong potential for school–health sector partnerships. Whole-school health models advocate exactly this kind of collaboration, where external experts supplement teacher knowledge and link classroom learning to community resources.^[52]^ Coordinated programs with input from both education and health authorities could turn fragmented awareness events into sustainable, curriculum-embedded interventions.

Although based on a small, nonrandom sample, this study should be interpreted as exploratory in nature, offering preliminary, context-specific insights into teacher perceptions of cancer education in Saudi schools. Overall, our findings suggest that while cancer education in Asir schools is currently ad hoc, there are early signals that merit further, more rigorous investigation. Several teachers emphasized the need for structured, consistent programs, aligning with global guidance that schools should integrate health promotion into routine teaching.^[27]^ By addressing the multilevel challenges identified by teachers, from Ministry mandates down to personal motivation, future programs could maximize their reach and impact. Finally, to build a stronger evidence base for school-based cancer education in Saudi middle and high schools, the next research phase must entail broader purposive sampling, inclusive of rural and private schools, audio-recorded interviews with systematic qualitative analysis, the inclusion of student and administrator perspectives, and a formal review of curriculum documents.

## Acknowledgments

The authors extend their appreciation to the Deanship of Research and Graduate Studies at King Khalid University for funding this work through Small Research Project (grant number RGP1/116/46).

## Author contributions

**Conceptualization:** Aziza Alshahrani, Zahrah Asiri.

**Data curation:** Aziza Alshahrani, Zahrah Asiri.

**Formal analysis:** Aziza Alshahrani, Zahrah Asiri.

**Funding acquisition:** Aziza Alshahrani.

**Investigation:** Aziza Alshahrani.

**Methodology:** Aziza Alshahrani, Zahrah Asiri.

**Project administration:** Aziza Alshahrani.

**Software:** Aziza Alshahrani.

**Supervision:** Aziza Alshahrani.

**Validation:** Aziza Alshahrani.

**Visualization:** Aziza Alshahrani.

**Resources:** Shahad Alahmari, Saja Alasmari, Malak Alsultan, Dina Alqahtani, Reem Alqahtani.

**Writing – original draft:** Shahad Alahmari, Saja Alasmari, Malak Alsultan, Dina Alqahtani, Reem Alqahtani.

**Writing – review & editing:** Shahad Alahmari, Saja Alasmari, Malak Alsultan, Dina Alqahtani, Reem Alqahtani.










