# Supplementary material for: Status of cancer education in middle and high schools in southern Saudi Arabia: An exploratory descriptive study
Source: Medicine (Baltimore). 2026 May 15;105(20):e48793. doi: 10.1097/MD.0000000000048793 (PMC13183015; doi:10.1097/MD.0000000000048793)
Supplement: Supplementary file 1 [file medi-105-e48793-s001.docx]

Supplementary Table 1. Teachers' responses describing their efforts to incorporate cancer education.

| Variable | Response Category | Teacher responses | Course | School level |
| --- | --- | --- | --- | --- |
| Do you personally incorporate cancer education into your teaching? | Curriculum-Based (covering only what is originally included in the curriculum) | Through the curriculum. | Biology | High |
|  |  | Some lessons and topics are related to this disease as part of educational objectives, and it is important to discuss and raise awareness about it. | Biology | High |
|  |  | Through the school curriculum. | Biology | High |
|  | Raising awareness about early detection | Raising students' awareness about early detection and simple screening methods that can be performed after completing the course. | Biology | High |
|  |  | Raising students' awareness about early detection and simple screening methods that can be performed after completing the course. | Science | Middle |
|  |  | Awareness and encouragement of early detection. | Science | Middle |
|  | Educating about cancer Risk Factors | About the harms of certain chemical compounds on the skin and their excessive use, which can lead to skin cancer and its widespread occurrence, along with other diseases. | Chemistry | High |
|  |  | How it develops and ways to prevent it. | Science | Middle |
|  |  | Raising awareness about the causes of increased cancer cases. | Science | Middle |
|  |  | Talking about food, smoking, and disease causes. | Science | Middle |
|  | Promoting general cancer awareness | Awareness presentations. | Chemistry | High |
|  |  | presentations and explanations of some diseases, discussions of students' research. | Biology | High |
|  |  | Providing a general idea about cancer. | Science | Middle |
|  |  | Sometimes, cancer is briefly addressed. | Chemistry | High |
|  |  | General information about the effects of gases and diseases. | Chemistry | High |
|  |  | The nature of the disease and how to detect it. | Biology | High |
|  |  | Through discussion | Science | Middle |
