# Supplementary material for: Status of cancer education in middle and high schools in southern Saudi Arabia: An exploratory descriptive study
Source: Medicine (Baltimore). 2026 May 15;105(20):e48793. doi: 10.1097/MD.0000000000048793 (PMC13183015; doi:10.1097/MD.0000000000048793)
Supplement: Supplementary file 2 [file medi-105-e48793-s002.docx]

Supplementary Table 2. Teachers' responses describing school efforts to include cancer awareness initiatives.

| Variable | Response Category | Teacher responses | Course | School level |
| --- | --- | --- | --- | --- |
| Does your school have any programs or initiatives that integrate cancer awareness into the curriculum? | Breast cancer awareness | Breast cancer | Chemistry | High |
|  |  | Lecture in collaboration with doctors about breast cancer and raising awareness | Science | Middle |
|  |  | Community partnership on breast cancer awareness | Chemistry | High |
|  |  | Breast cancer awareness campaigns from health guidance services in school | Biology | High |
|  |  | Breast cancer initiative | Biology | High |
|  |  | Awareness about breast cancer only | Chemistry | High |
|  | General educational activity and community partnership | Community partnership with a doctor who comes to the school to give a lecture on this topic Activities | Biology | High |
|  |  | Holding seminars at the school by female doctors for students | Biology | High |
|  |  | Activities | Physics | High |
|  |  | Holding seminars at the school by female doctors for students | Biology | High |
|  |  | Community partnership with the neighborhood care center and some private clinics | Chemistry | High |
|  |  | Health guidance, doctors, radio programs, and World Cancer Day for all types of cancer | Chemistry | High |
|  |  | Programs and activities | Science | Middle |
|  |  | Raising students' awareness of disease symptoms | Biology | High |
|  |  | School broadcasts | Science | Middle |
