# Supplementary material for: Status of cancer education in middle and high schools in southern Saudi Arabia: An exploratory descriptive study
Source: Medicine (Baltimore). 2026 May 15;105(20):e48793. doi: 10.1097/MD.0000000000048793 (PMC13183015; doi:10.1097/MD.0000000000048793)
Supplement: Supplementary file 3 [file medi-105-e48793-s003.docx]

Supplementary Table 3. Teachers' responses explaining the lack of training or professional development on teaching cancer-related topics.

| Variables | Response Category | Teacher responses | Course | School level |
| --- | --- | --- | --- | --- |
| Have you ever received training or professional development on teaching topics related to cancer? | Lack of Coordination or Institutional Support | Lectures in cooperation with doctors about breast cancer and awareness campaigns. | Chemistry | High |
|  |  | It is not required by the Ministry of Education and not a mandatory condition | Biology | High |
|  |  | I was not nominated by my supervisor for such programs | Natural sciences | Middle |
|  | Lack of Opportunity or Access | I didn’t get the opportunity | Science | Middle |
|  |  | I haven’t found a chance for that. | Science | Middle |
|  |  | I didn’t get the opportunity to access courses or curricula | Chemistry | High |
|  | Lack of Resources | The training and workshops are on other topics, not cancer | Biology | High |
|  |  | No one is available to deliver such lesson. | Physics | High |
|  |  | No available programs | Biology | High |
|  |  | I don’t know; it’s not my specialty or the specialty of the person in charge | Biology | High |
|  | Not Within Subject Scope | It’s not within my field | Science | Middle |
|  |  | Because it's not part of my specialty | Biology | High |
|  |  | Because the disease is not in my area of specialization | Chemistry | High |
|  | Low personal interest | Not interested | Earth and space science | High |
|  | Perceived sufficiency of existing curriculum | It is already covered in the course, and therefore, we just teach them what we have. | Biology | High |
