# Supplementary material for: Status of cancer education in middle and high schools in southern Saudi Arabia: An exploratory descriptive study
Source: Medicine (Baltimore). 2026 May 15;105(20):e48793. doi: 10.1097/MD.0000000000048793 (PMC13183015; doi:10.1097/MD.0000000000048793)
Supplement: Supplementary file 4 [file medi-105-e48793-s004.docx]

Supplementary Table 4. Teachers' responses describing why they believe cancer education in schools would be beneficial.

| Variable | Theme | Teacher responses | Course | School level |
| --- | --- | --- | --- | --- |
| Do you believe cancer education in schools would be beneficial? | Promoting Basic Cancer Knowledge | To learn about the types of cancer, educate students, and understand its dangers. | Chemistry | High |
|  |  | To raise awareness. | Biology | High |
|  |  | To educate students about this disease. | Biology | High |
|  |  | To educate students about this disease. | Biology | High |
|  |  | To raise awareness among students and also their families through the information provided by the school. | Science | Middle |
|  | Promoting Prevention and Early Detection Awareness | To ensure the next generation is aware and capable of detecting issues early before they worsen in the body. | chemistry | High |
|  |  | Students gain knowledge about prevention and disease detection. | Biology | High |
|  |  | Students’ lifestyles are unhealthy, with reliance on processed foods and a lack of proper care. | Chemistry | High |
|  |  | Early detection | Biology | High |
