# Supplementary material for: Status of cancer education in middle and high schools in southern Saudi Arabia: An exploratory descriptive study
Source: Medicine (Baltimore). 2026 May 15;105(20):e48793. doi: 10.1097/MD.0000000000048793 (PMC13183015; doi:10.1097/MD.0000000000048793)
Supplement: Supplementary file 5 [file medi-105-e48793-s005.docx]

Supplementary Table 5. Teachers’ reasons supporting the feasibility of incorporating cancer education into the school curriculum.

| Variable | Response Category | Teachers’ response | Course | School level |
| --- | --- | --- | --- | --- |
| Why do you think integrating cancer topics into the curriculum would be possible within your classroom or school? | Incorporating Cancer in the Curriculum | Because it is already included in the curriculum | Biology | High |
|  |  | Some topics already cover mutations in cell division | Science | Middle |
|  |  | It is already part of science curricula | Science | Middle |
|  |  | Related to biology | Science | Middle |
|  |  | Due to the presence of related lessons in the curriculum | Chemistry | High |
|  |  | Because students learn about body systems and we touch upon cancer | Chemistry | High |
|  | Raising Awareness and Health Education | To educate and raise awareness about diseases | Chemistry | High |
|  |  | Increase students’ awareness and enthusiasm to explore more | Science | Middle |
|  |  | For Awareness | Biology | High |
|  |  | Because health should always be discussed | Chemistry | High |
|  | Community Benefit and Prevention | For a healthier society | Chemistry | High |
|  |  | To prevent it in the future | Science | Middle |
|  | Understanding the Disease and Its Causes | To understand its causes | Science | Middle |
|  |  | More information about the disease that benefits the students | Biology | High |
